# Design and Study of a Photo-Switchable Polymeric System in the Presence of ZnS Nanoparticles under the Influence of UV Light Irradiation

**DOI:** 10.3390/polym14050945

**Published:** 2022-02-26

**Authors:** Guadalupe del C. Pizarro, Wilson Alavia, Karen González, Héctor Díaz, Oscar G. Marambio, Rudy Martin-Trasanco, Julio Sánchez, Diego P. Oyarzún, Andrónico Neira-Carrillo

**Affiliations:** 1 Departamento de Química, Facultad de Ciencias Naturales, Matemáticas y Medio Ambiente, Universidad Tecnológica Metropolitana, Santiago 7800003, Chile; w.alaviam@outlook.com (W.A.); karen.005@hotmail.es (K.G.); hdiaz@utem.cl (H.D.); omarambi@utem.cl (O.G.M.); rudy.martint@utem.cl (R.M.-T.); 2 Programa Institucional de Fomento a la Investigación, Desarrollo e Innovación (PIDi), Universidad Tecnológica Metropolitana, Santiago 7800003, Chile; 3 Departamento de Ciencias del Ambiente, Facultad de Química y Biología, Universidad de Santiago de Chile, USACH, Santiago 9170022, Chile; julio.sanchez@usach.cl; 4 Laboratorio de Nanotecnología, Recursos Naturales y Sistemas Complejos, Facultad de Ciencias Naturales, Universidad de Atacama, Copiapó 1530000, Chile; diequim@gmail.com; 5 Departamento de Ciencias Biológicas, Facultad de Ciencias Veterinarias y Pecuarias, Universidad de Chile, Santiago 7800003, Chile; aneira@uchile.cl

**Keywords:** light-sensitive materials, spiropyrans, photoactive polymeric system, morphological surface characteristic, photo-switchable properties

## Abstract

Recent progress in the field of photosensitive materials has prompted a need to develop efficient methods to synthesize materials with basic intermolecular architectural designs and novel properties. Accordingly, in this work we design and study a photoactive polymer as a photo-switchable polymeric system in the presence and absence of ZnS nanoparticles (average size < 10 nm) at 5 wt.%. The influence of UV light irradiation on its properties were also studied. The photoactive block copolymer was obtained from styrene (S) and methyl methacrylate (MMA) as monomers and 1-(2-hydroxyethyl)-3,3-dimethylindoline-6-nitrobenzopyran (*SP*) was grafted to the block copolymer backbone as a photochromic agent. Furthermore, the incorporation of ZnS (NPs) as photo-optical switch component into the system enhances the purple colored photo-emission, with the open form of the spiropyran derivative (merocyanine, MC). The ZnS stabilize the isomeric equilibrium in the *MC* interconversion of the photochromic agent. The photo-switchable properties of the PS-*b*-PMMA-*SP* in the presence of ZnS (NPs) were examined using UV-VIS spectroscopy, Photoluminescence (PL) spectroscopy, optical fluorescence and scanning electronic microscopy (SEM-EDX.). The observed changes in the absorbance, fluorescence and morphology of the system were associated to the reversible interconversion of the two states of the photochromic agent which regulates the radiative deactivation of the luminescent ZnS NPs component. After UV irradiation the photoactive polymer becomes purple in color. Therefore, these basic studies can lead to the development of innovative functional and nanostructured materials with photosensitive character as photosensitive molecular switches.

## 1. Introduction

The combination of polymers with nanoparticles is a useful strategy for designing novel functional materials [1,2] and a wide variety of methods can be explored for their production such as electrospinning [3], electrospraying [4] and traditional chemical and physical deposition methods.

In the literature, several inorganic nanoparticles (NPs) such as SiO_2_ [5,6], ZnO, [7,8], ZnS [9], Ag [10,11,12], CuO [13,14], MoS_2_ [15] and TiO_2_ [16,17,18] have been shown to be useful for the preparation of functional materials [19,20,21,22]. On the other hand, the establishment of multiple functions requires the combination of hydrophobic surfaces with micro/nanoscale structures, low surface energy and auxiliary components [23,24,25,26,27]. In addition to traditional processing methods, researchers have proposed several strategies, such as layer-by-layer assembly [28], dip coating [29], chemical vapor deposition [30] and etching [29,30,31] as routes to obtain the desired functionality.

Due to their low cytotoxicity, ZnS are very interesting NPs to explore with respect to the preparation and properties of such composites. Kole et al. [32] synthesized ZnS quantum dots (QDs) and ZnS/PMMA nanocomposites and determined their morphological and optical properties, and thermal stability. It was found that the interaction of the ZnS NPs with the polymer matrix is weak and that the incorporation of the NPs into the polymer changes its transmittance from 86% to 45% in the visible region.

Moreover, Nayak et al. [9] prepared ZnS/PMMA nanocomposites and reported their optical, electrical and dielectric properties. The nanocomposite morphology, that combined a pebbled and nanosphere like structure, exhibited a bandgap of 3.30 eV and its optical spectra showed blue, green and yellow absorption bands. The nanocomposite PMMA-ZnS (5 wt.%) showed optimal luminescence with a decay time of 4.8413 ns and a color purity of 34.08%. These characteristics make ZnS/PMMA appropriate for use as an emissive layer in organic light-emitting diode (OLED or organic LED) devices. On the other hand, Pizarro et al. synthesized ZnS nanoparticles (diameter size < 10 nm) into the polymer matrix obtained from the self-assembled poly(acrylic acid)-block-poly(N-phenylmaleimide) using an Atom Transfer Radical Polimerization (ATRP) method [33].

In terms of the physical mechanism of such devices, photo-switchable moiety can be grafted to a polymer backbone or incorporated into it to produce different observable effects. In the latter, the incorporated photo-switchable unit can also affect the nature of the polymer backbone itself [34]. The nature of the polymer backbone can also influence the kinetics of the open/closed ring isomerization of the photoactive units, affecting the stability, or even leading to photo degradation [35,36,37,38]. The exploration of such light-responsive molecules in devices typically requires immobilization on the surface through an appended functionality that does not interfere with the light switching behavior. This has been achieved for photo-switchable molecules using formations of self-assembled monolayers (SAMs) [39,40,41,42], bilayers [43,44,45] and incorporation into polymer films [46,47,48,49], beads and nanoparticles [50,51,52]. Furthermore, the polymerization process itself must be compatible with the photo/electroactive switching units, and in addition, the incorporation of the photoresponsive functionality must not affect the polymerization.

On the other hand, a photo-switchable molecule is defined as a reversible photo-induced transformation of a chemical species between two forms each displaying different optical properties. Although organic photochromic compounds have been the subject of intensive work over recent decades, they still await major commercial exploration. The change produced in the chemical structure of the species often allows it to absorb in its excited state (color) in a certain region of the spectrum UV-Vis, generally in the visible region, returning to its basal state (colorless) in response to a second radiation, usually in the visible spectrum or induced thermally [53,54,55]. A novel application of these compounds is with the design and manufacture of variable transmission optical materials [56,57,58,59]. These changes to the molecular properties can also be applied to various photonic devices, such as erasable optical memory media and photo-optical switch components [60,61,62,63,64,65].

This work reports the preparation and characterization of a photoactive polymer as a photo-switchable polymeric system in the presence and absence of ZnS nanoparticles. The system was also studied under the influence of UV light irradiation and its affect upon the system containing fluorescent ZnS NPs as well as its optical and morphological properties. Therefore, this work can contribute to the development of innovative functional and nanostructured materials with photosensitive character, since photosensitive molecular switches can be applicable in the development of thermal and optical technological devices.

## 2. Materials and Methods

Chemicals were used without further purification and were of analytical grade. Methyl methacrylate (MMA) (*M*_w_: 100.1 g mol^−1^; 99%, Sigma-Aldrich, St. Louis, MO, USA), Benzoyl peroxide (BPO, *M*_w_: 242.2 g mol^−1^; 99.98%, Sigma-Aldrich); Copper (I) bromide (CuBr, *M*_w_: 143.45 g mol^−1^, 99.99%, Sigma-Aldrich); 2,2′-Bipyridine (Bpy, *M*_w_: 156.1 g mol^−1^; ≥99%, Sigma-Aldrich); 1,3,3-Trimethyl-2-methylen-indoline (*M*_w_: 173.2 g mol^−1^, 97%, Sigma-Aldrich); 2-hydroxy-5-nitrobenzaldehyde (Mw: 167.12 g mol^−1^, 98% Sigma-Aldrich). 2-bromoethanol (C_2_H_5_BrO, Sigma-Aldrich) and 2-butanone (C_2_H_5_COCH_3_, 99.0%, Sigma-Aldrich).

### 2.1. Measurement

Instrumentation and Equipment: Fourier-transform infrared FT-IR spectra were recorded using a Bruker Vector 22 (Bruker Optics GmbH, Inc., Ettlingen, Germany). The absorption spectra of the films were recorded at 25 °C between 250–700 nm using a Perkin Elmer Lambda 35 spectrophotometer. Photoluminescence (PL) measurements were performed at room temperature by a fluorescence spectrometer system (Perkin Elmer, Cambridge, MA, USA, model L 55), using an excitation wavelength of 320 nm. The molecular weights and polydispersity (*M*_w_/*M*_n_) were determined by size exclusion chromatography (SEC) using a Shimatzu LC 20 instrument equipped with RI detectors with dimethylformamide (DMF) as the solvent (flow rate: 1.0 mL/min). Measurements were performed at 30 °C and polystyrene standard was used for the calibration.

The UV-Vis absorption and fluorescence emission spectra of dilute copolymer solutions were recorded at room temperature. The samples were dissolved in acetonitrile and cast on to glass substrate. Then, the morphological properties of the photoactive block copolymer and for the photoactive polymer functionalized with ZnS surface films were analyzed using optical fluorescence microscopy and scanning electron microscopy (SEM) with energy dispersive X-ray spectroscopy (SEM-EDX), using a scanning electron microscope EVO MA 10, Zeiss with an EDX penta FET precision detector Oxford instruments X-act of the Center for the Development of Nanoscience and Nanotechnology, Chile.

### 2.2. Preparation of ZnS Nanoparticles

First, 2.39 g of Zn(COOCH_3_)_2_·2H_2_O was dissolved in 10 mL of THF to reach a concentration of 0.13 M. Subsequently, the pH of the solution was adjusted to 6.0 via dropwise addition of a solution of 1 M of NaOH. The conversion of Zn^2+^ to ZnS was achieved by dropping an equimolar solution of Na_2_S (1 g in 10 mL of THF) into the stirring solution. Observing the XRD spectra, the XRD pattern of ZnS blende shows the one broad feature around 26°, which is formed by the overlap of the (100), (002) and (101) line reflections. Then, the XRD patterns of ZnS exhibited a broadening and a shift to higher angles of the first line diffraction feature, initially located around 36°. Subsequently, a peak of a relative intensity was observed around 47° which confirmed the crystalline structure of ZnS (Zinc blende) cubic lattice. Moreover, the appearance of a new peak around 56° was observed. These peaks could easily be assigned to the planes (111), (220) and (311) respectively of the cubic phase. The average crystallite size was calculated as < 10 nm using Scherrer’s equation. *D* = *kλ/β cos(θ)* where D is the crystallite size, *k* is a constant (0.9 for the spherical shape), *λ* is the wavelength of the X-ray radiation, *β* is the line width obtained after correction for instrumental broadening and *θ* is the angle of diffraction [33]. The XRD patterns of ZnS are shown in Figure S1 (see Supplemental files).

### 2.3. Synthesis and Characterization of the Block Copolymer

Polystyrene-block-polymethylmethacrylate (PS-*b*-PMMA) was obtained via the ATRP technique using a narrow polydispersity index (D = 1.2) and average molecular weight (*M*_w_) of 24.8 kDa. The block copolymer was obtained using polystyrene (PS) macroinitiator/CuBr/BPy as the catalyst system and methylmethacrylate (MMA) as co-monomer in a molar ratio of 1/1/2/100. The resulting block copolymer was obtained as a white powder (yield: 85%). The process above is described in more detail elsewhere [66,67]. The FT-IR spectrum exhibited characteristic absorption bands at 3066–3026 cm^−1^ [υ(CH, aromatic)]; 2923–2852 cm^−1^ [υ(CH, CH_2_)]; 1948–1875 cm^−1^ [υ(aromatic overtone)]; 1650.34 cm^−1^ [υ(–C=O from MMA)]; 758.31 and 691.26 cm^−1^ [υ(aromatic ring)]. The FT-IR spectrum exhibited weak absorption bands at 3066–3026 cm^−1^ [υ(CH, aromatic)]; 2923–2852 cm^−1^ [υ(CH, CH_2_)]; at 1618 (stretching –C=O, ester); 1292 and 1177 (symmetric and asymmetric alkyl ester stretching), at 1490 cm^−1^, corresponding to the C=C stretching of the aromatic ring, 758.31 and 691.26 cm^−1^ [υ(aromatic ring)].

### 2.4. Synthesis and Characterization of 1-(2-Hydroxyethyl)-3,3-dimethylindoline-6-nitrobenzopyran as Photochromic Component

In a first step, the synthesis of the 1-(2-hydroxyethyl)-2,3,3-trimethylindolenine bromide was carried out in a schlenk tube in which 4.0 mL (3968 mg, 25 mmol) of 2,3,3-trimethylindolenine were added, then 1.8 mL (3173 mg, 25 mmol) of 2-bromoethanol in 3.15 mL (2536 mg, 35 mmol) of 2-butanone was used as solvent. Subsequently, the mixture was degassed in an inert atmosphere, frozen with liquid nitrogen, then several cycles of vacuum and thawing were performed. The synthesis tube was placed in an oil bath at 140 °C with constant stirring for 10 h, then at room temperature the mixture was filtered to obtain a pink solid. This was purified by extraction in a benzene soxhlet for 24 h, until the solution became colorless. The synthesis reaction is shown in Figure S2.

In the second step, 1-(2-hydroxyethyl)-3,3-dimethylindoline-6-nitrobenzopyran was obtained. In a 250 mL three-neck rounded bottom flask in an oil bath equipped with a magnetic stirrer and condenser, 2 g (7.04 mmol) of 1-(2-hydroxyethyl)-2,3,3-trimethylindolenine bromide was added and, subsequently, 1.2 g (7.04 mmol) of 2-hydroxy-5-nitrobenzaldehyde (C_7_H_5_NO_4_, 98%, Sigma-Aldrich, St. Louis, MO, USA) was added, in addition to 2 mL of trimethylamine (C_3_H_9_N, 99% Sigma-Aldrich) (2680 mg, 22.7 mmol) in 20 mL of ethanol. All were heated until boiling (78 °C) and maintained for 4 h. After, the solution was allowed to cool and the ethanol was evaporated using a rotary evaporator. The product was extracted in a separator funnel with a solution of 10% HCl and chloroform in equal volumes to recover the organic phase where the product is located. After this, the product was dried in the presence of magnesium sulfate, filtered and the chloroform evaporated. The products obtained were purple crystals, yield: 75%, (The main synthesis reaction is shown in Figure S3). The characteristic signals of the photochromic compound 1-(2-hydroxyethyl)-3,3-dimethylindoline-6- nitrobenzo pyran by ^1^H-NMR and FT-IR spectrum are shown in Figure S4.

### 2.5. Photoactive Copolymer with SP Photochromic Compound Grafted to the Lateral Chain

In a 250 mL three-neck round bottom flask in an oil bath, equipped with a magnetic stirrer and condenser, 0.2 g (0.0036 mmol) of copolymer PS-*b*-PMMA, 0.0013 g (0.0036 mmol) of 1-(2-hydroxyethyl)-3,3-dimethylindoline-6-nitrobenzopyran and 1 drop of H_2_SO_4_ in 5 mL of tetrahydrofuran were heated for 4 h. The obtained product was a yellow powder whose yield was 80%. The following vibrational bands were observed in the FT-IR spectra υ (cm^−1^, KBr): a weak band at 3460; 1618 (stretching –C=O, ester); 1292 and 1177 (symmetric and asymmetric alkyl ester stretching). Typical broad signals of carboxylic acid and hydroxyl group are observed but with a low intensity; therefore, it was possible that the polymer was not fully functionalized with the photochromic compound.

**Figure 1:**
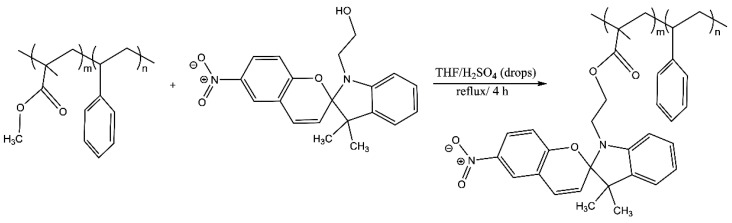
Schematic representation of the (PS-*b*-PMMA-*SP*) block copolymer functionalized with photochromic agent (*SP*).

Figure 1 shows the copolymer block functionalized with the photochromic compound (PS-*b*-PMMA-*SP*) after being irradiated by ultraviolet light changing from a colorless ground state (closed) to a colored excited state (open).

### 2.6. Measurement by UV-Visible Spectrophotometry

A certain amount of copolymer functionalized with photochromic compound was prepared using acetonitrile as a solvent (0.5 g/L); 2 mL of this solution was taken and deposited in a quartz cell to record the corresponding UV-Vis spectrum. The colored state of the compound in solution was reached after irradiating with 365 nm (peak wavelength) for 5 min. The photogenerated species reverted to its original form after light irradiation, restoring the original absorption spectrum.

### 2.7. Characterization of SP by UV-Visible Spectrophotometry

**Figure 2:**
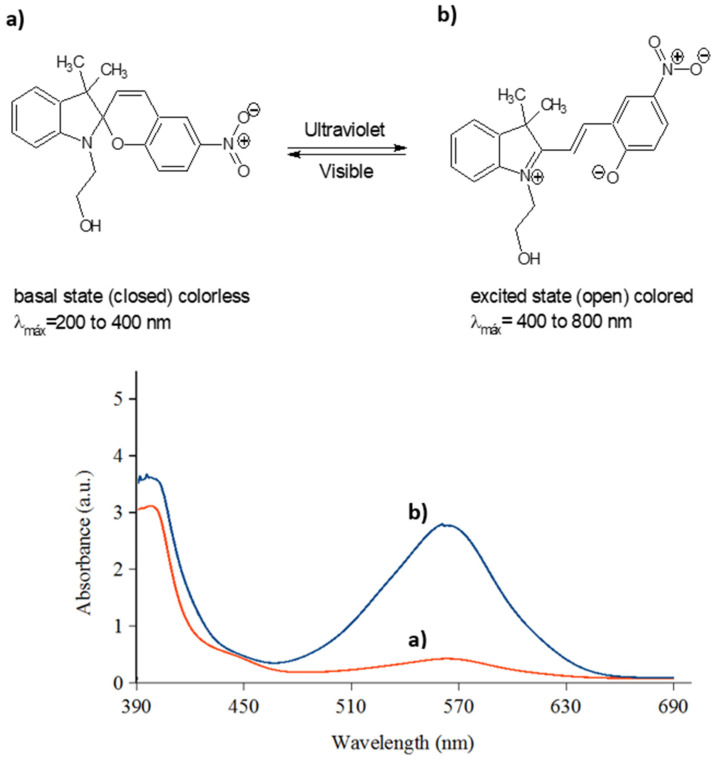
Schematic representation of photo induced reversible interconversion of 1-(2-hydroxyethyl)-3,3-dimethylindoline-6-nitrobenzopyran (*SP*) (**a**) before UV-Vis light irradiation, (**b**) after light irradiation.

As can be seen in Figure 2, the 1-(2-hydroxyethyl)-3,3-dimethylindoline-6-nitrobenzopyran (*SP*) compound after irradiation by ultraviolet light changes from its ground state (closed ring, colorless) to the excited state (open ring, colored, with purple color) resulting in the formation of the merocyanine (*MC*) compound. The compounds in solution were irradiated with an ultraviolet light lamp that emits in a range of 250 to 380 nm (peak wavelength 365 nm) with an intensity of 95 mW/cm^2^, for 5 min.

As can be seen in Figure 2a, before ultraviolet irradiation the photochromic compound shows UV-Vis absorbance bands located around 384 nm, which is in the ultraviolet region of the spectrum. After irradiating, Figure 2b shows a new absorption peak at a *λ* max of 570 nm. The appearance of a new band in the visible region was attributed to the great resonance effect that the nitro group has in the open state of the molecule, where the activation of an energy transfer process was induced in the photochromic compound upon ultraviolet irradiation [68]. As a result, the photoinduced bathochromic shift of the photochrome absorption was obtained.

### 2.8. Photoactive PS-b-PMMA-SP Functionalized with ZnS NPs

The photoactive block copolymer (5 mg) was dissolved in 1 mL of acetonitrile to reach a concentration of 0.2% (*W*/*V*). Subsequently, the ZnS (NPs) (average size < 10 nm) were incorporated into solution in a concentration of 5 wt.%. The solution was left to stir vigorously for 2 h to guarantee the coordination of the nitro groups of the PS-*b*-PMMA-*SP* to the ZnS on the nanoparticle surface.

## 3. Results and Discussions

### 3.1. Chemical and Morphological Characterization

**Figure 3:**
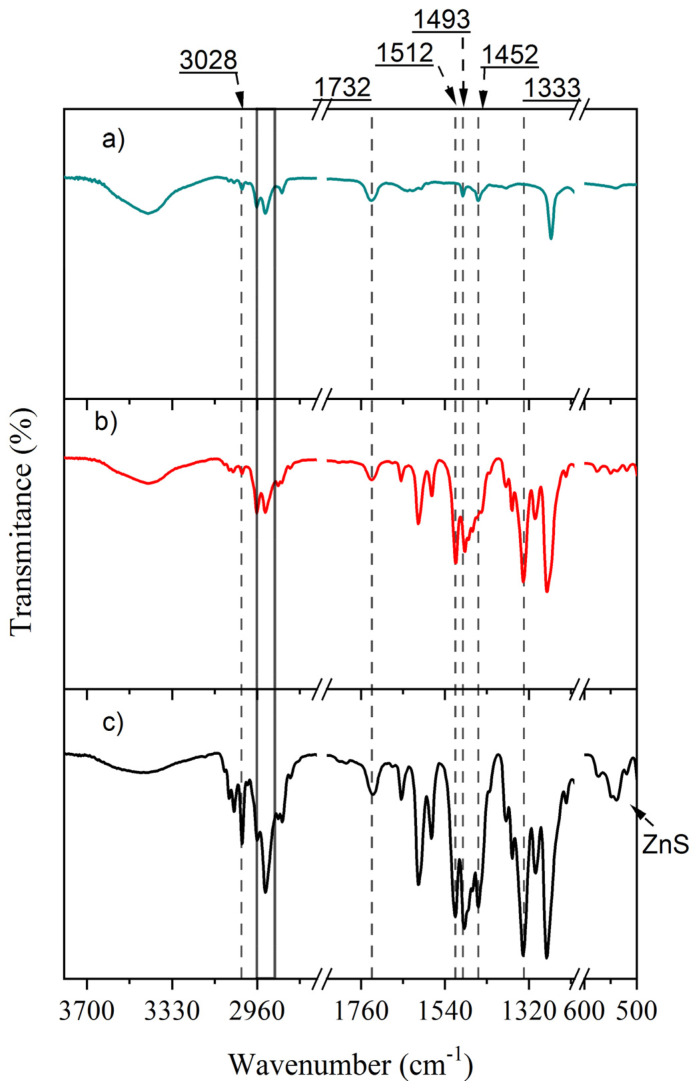
FT-IR spectrum of (**a**) PS-*b*-PMMA, (**b**) PS-*b*-PMMA-*SP* and (**c**) PS-*b*-PMMA-*SP*-ZnS functionalized block copolymer. The *x*-axis in the spectrum appears bracket for the sake of simplicity.

The FT-IR spectra for PS-*b*-PMMA, PS-*b*-PMMA-*SP* and PS-*b*-PMMA-*SP*-ZnS functionalized block copolymers are shown in Figure 3.

The FT-IR spectrum of the PS-*b*-PMMA block polymer shows the typical signals of carbonyl stretching from ester at 1732 cm^−1^ and the phenyl C=C stretching from the aromatic ring at 1493 and 1452 cm^−1^. The signal from the antisymmetric and symmetric –C–H stretching of the methylene group at the polymer backbone is observed at 2962 and 2928 cm^−1^, respectively. From the phenyl ring this band is observed at 3028 cm^−1^.

Three important changes are observed when comparing this with the spectrum of the photoactive compound grafted to the polymer backbone (Figure 3b): (i) an increase in the intensity of the –C=C– stretching modes of the aromatic ring with respect to the aliphatic –C–H stretching, (ii) the presence of two intense bands at 1512 and 1333 cm^−1^ corresponding to the antisymmetric and symmetric –NO_2_ stretching, respectively and (iii) the band at 1452 cm^−1^ is recorded as a shoulder. Regarding the latter, it could suggest that the *SP* molecule is in its spiro form and restricts one of the –C=C– vibration modes. These features indicate that the grafting of the photoactive molecule has taken place.

In the presence of the ZnS NPs (Figure 3c) the spectrum shows a signal at 536 cm^−1^, characteristic of these nanoparticles. Additionally, an increase in the intensity ratio of –C–H stretching of methine at 3028 cm^−1^ with respect to the –C–H in the methylene at 2926 is observed. Another interesting feature is the recording of the aromatic –C–H stretching, as a peak, at 1452 cm^−1^. These two later features could be indicative of the opening of the rigid *SP* structure into the more relaxed merocyanine form, due to its interaction with the nanoparticle surface.

**Figure 4:**
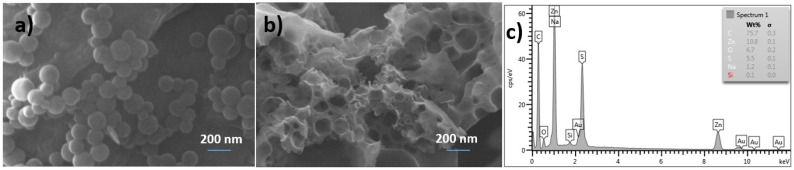
SEM micrographs of (**a**) PS-*b*-PMMA-*SP* and (**b**) PS-*b*-PMMA-*SP* composite with ZnS NPs and (**c**) EDX analysis of the composite polymer.

The morphology and composition of the photoactive polymer and its corresponding composite with ZnS NPs at 5 wt.%. were determined by SEM and EDX analysis (Figure 4). The samples were prepared by using the solvent-assisted technique.

The micrograph PS-*b*-PMMA-*SP* (Figure 4a) shows a spherical-like morphology with an average diameter of 200 nm which are characteristics of block copolymers due to the trend to self-assemble. As can be noted, in the presence of ZnS NPs, the spherical-like structures of polymer are absent (Figure 4b). This could be indicative of the interaction between the block copolymer with the ZnS NPs hindering the self-assembling of the polymer. The EDX spectrum inset in Figure 4b shows the elemental composition of the corresponding image. Additionally, to the common lines of carbon and oxygen from the polymer, other lines at 1.0 and 8.6 keV corresponding to Zinc and at 2.2 keV corresponding to Sulphur in the nanoparticles are observed. The micrographs in films show that the inorganic part is well dispersed in the photoactive polymer system and there is a uniform and homogeneous distribution of ZnS NPs in the phases.

### 3.2. Optical Pproperties: UV-Vis Spectrophotometry

**Figure 5:**
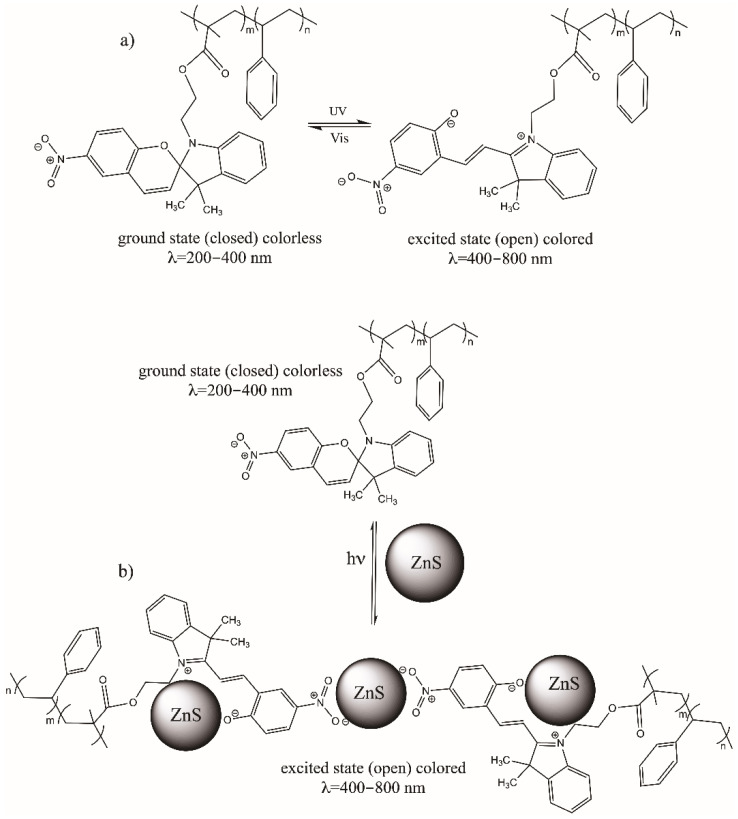
Photochromic reaction of polymer-functionalized irradiated with an ultraviolet light lamp at 365 nm (95 mW/cm^2^) for 5 min, (**a**) PS-*b*-PMMA-*SP* and (**b**) its interaction with ZnS (NPs).

Figure 5 shows a schematic mechanism of the reaction of the photoactive polymer (a) without ZnS (NPs) and (b) with ZnS (NPs), both before and after irradiation. As indicated in the diagram, the excitation energy of the ZnS NPs can be transferred to the photogenerated state of the photochromic compound, if the two components are sufficiently close to each other. Thus, the emission of these compounds can be repeatedly turned on and off simply by switching their photochromic component back and forth between these two states in response to optical stimulation [68].

**Figure 6:**
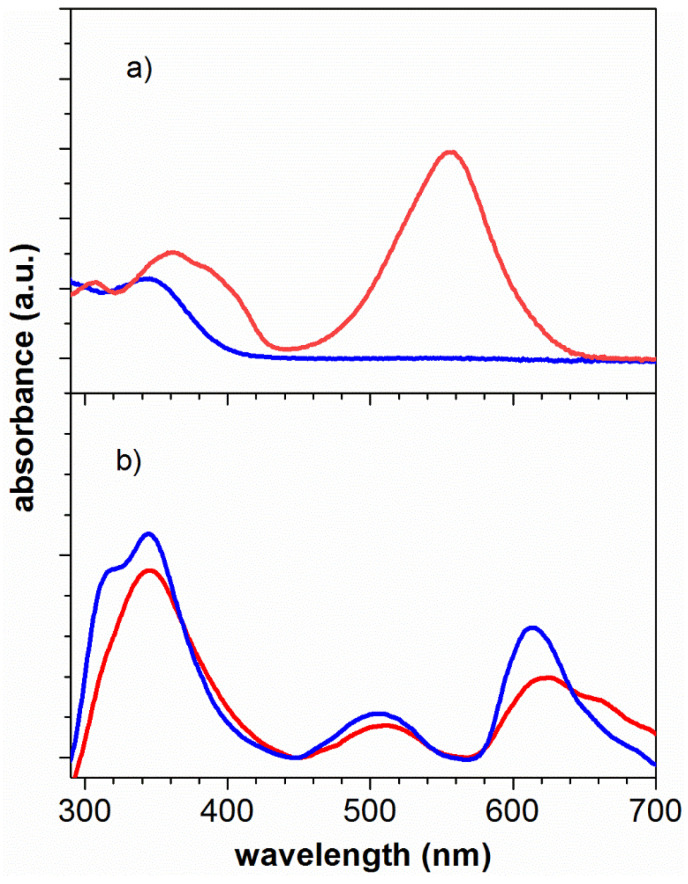
Ultraviolet–visible spectra of an acetonitrile solution of: (**a**) PS-*b*-PMMA-*SP* after irradiated (red spectrum) and (**b**) PS-*b*-PMMA-*SP*-ZnSNPs at 5 wt.%, after irradiated for 5 min (red spectrum) and 10 min (blue spectrum) with an ultraviolet light lamp at 365 nm (95 mW/cm^2^).

Figure 6 shows the absorbance spectra before and after UV-irradiation (365 nm) for (a) P(S)-*b*-P(MMA)-*SP* and P(S)-*b*-P(MMA)-*SP*-ZnS (NPs) for 5 min and 10 min. The most noticeable change of absorbance is within the wavelength range between 450 and 650 nm and between 570 and 700 nm, respectively. In Figure 6a after irradiation for 5 and 10 min, there appears a band at 550 nm (red color) which corresponds to the *SP* moiety in the ring-opened isomer form. In Figure 6b new bands at 620 nm were observed which correspond to the *SP* moiety in the ring-opened state after UV-irradiation at 365 nm for 5 min and 10 min with ZnS (NPs). As can be noted, after irradiation, the functionalized block copolymer switches reversibly between two states as identified by the different absorption features in the visible region.

The addition of the ZnS (NPs) to the photoactive polymer resulted in an absorbance band at 620 nm compared to the photoactive polymer alone. This could be attributed to embodiment of ZnS (NPs) into PS-*b*-PMAA-*SP* polymeric matrix [9]. As expected, the photochromic opening reaction is thermodynamically feasible, and its response depends on the physical state of the system [69].

### 3.3. Fluorescence Studies: PL Spectroscopy

**Figure 7:**
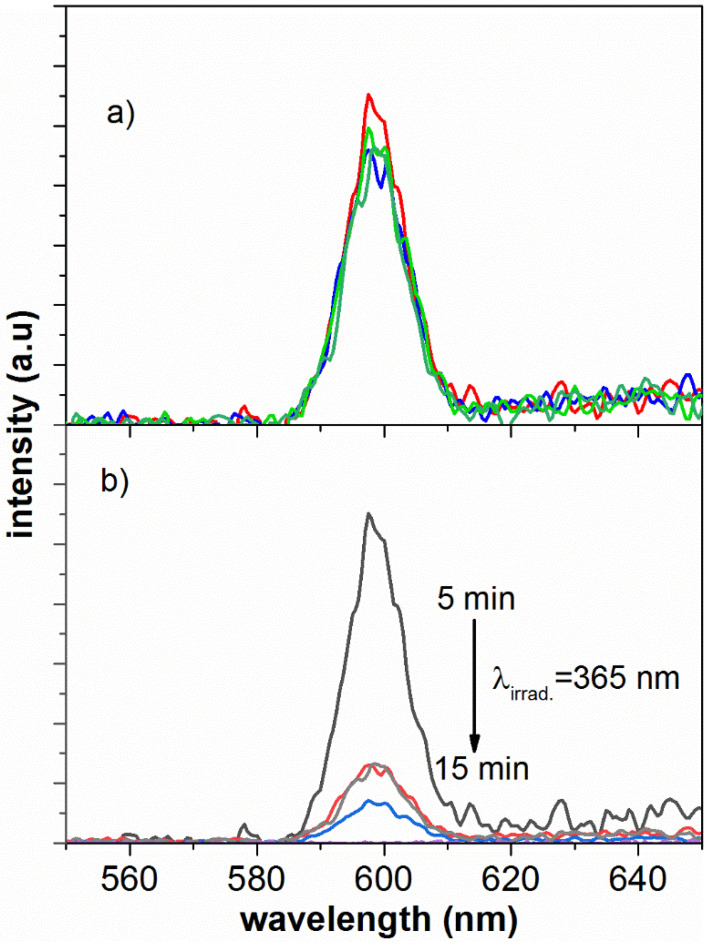
Emission spectrum of an acetonitrile solution of PS-*b*-PMMA-*SP* (**a**) 5, 10 and 15 min after ultraviolet irradiation and (**b**) PS-*b*-PMMA-*SP*-ZnS (NPs) (*λ_Ex_* = 320 nm) after ultraviolet irradiation (365 nm, 0.4 mW cm^−2^ for 5 min, photoactive polymer alone (black line) and PS-*b*-PMMA-*SP*-ZnS NPs for 5, 10 and 15 min after ultraviolet irradiation (c = 5% *w*/*w*).

Figure 7 shows the emission signals for an acetonitrile solution of the photoactive polymer after 5, 10 and 15 min of UV irradiation (a) PS-*b*-PMMA-*SP* (1.2 mg mL^−1^) after 5 min UV irradiation and (b) PS-*b*-PMMA-*SP*-ZnS(NPs) (*λ*_Ex_ = 320 nm) after 5, 10 and 15 min of UV irradiation (365 nm, 0.5 mW/cm^2^). After irradiation, the photoactive polymer (PS-*b*-PMMA-*SP*) became purple in color, a characteristic of the merocyanine (*MC*) formation, in accordance with the concomitant appearance of an absorption band at 590 nm. This emission is independent of the irradiating time for the PS-*b*-PMMA-*SP* (Figure 7a). Indeed, this ZnS fluorophore emits at 559 nm in acetonitrile, whereas the *SP* spiropyran derivate does not absorb. Upon ultraviolet irradiation, the colorless *SP* spiropyran switches to the colored merocyanine with the concomitant appearance of an intense absorption band mentioned. The overlap between the absorption of the ZnS fluorophore and the emission of merocyanine indicates that the electron transition from the photoexcited MC to ZnS is electron-donor mediated.

In this way, the emission band of the polymer in the presence of nanoparticles (PS-*b*-PMMA-*SP*-ZnS(NPs) stabilizes the *MC* isomer of the photochromic agent; this effect is independent of the exposure irradiation time (Figure 7b).

The effect of ZnS nanoparticles on the photoluminescent properties of the photochromic pendant moiety can be explained in terms of energy transference. The band of the polymer at 600 nm overlaps with the emission band of the excited ZnS (NPs) until the photogenerated state of the photochromic moiety. Therefore, the excitation energy of the ZnS (NPs) can be transferred to the photogenerated state of the photoactive polymer when both components are sufficiently close to one each other. In addition, this transformation can encourage the transfer of one electron from the excited ZnS(NPs) to the photogenerated state of the photoactive polymer. Consistently, the emission spectrum of the PS-*b*-PMMA-*SP*-ZnS(NPs) shows a pronounced decrease in the luminescence at 600 nm, upon ultraviolet irradiation, because of the transformation *MC*↔*SP* of the photochromic ligand [9].

### 3.4. Characterization by Optical Fluorescence Microscope

**Figure 8:**
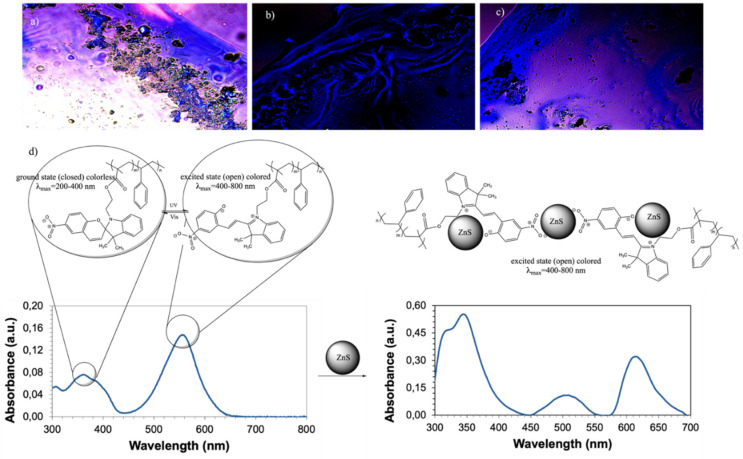
Optical fluorescence images (in CS_2_) of the photoactive polymer-*SP* prepared at 3 g L^−1^ (**a**) without ultraviolet irradiation under natural light; (**b**) with ultraviolet irradiation under fluorescence; (**c**) with ZnS (NPs) 5 wt.% under ultraviolet irradiation at 365 nm and under fluorescence (**d**) Ultraviolet–visible spectra of an acetonitrile solution of PS-*b*-PMMA-*SP* after irradiated and PS-*b*-PMMA-*SP*-ZnS NPs at 5 wt.%, after irradiated for 10 min with an ultraviolet light lamp at 365 nm (95 mW/cm^2^).

Figure 8 shows optical microscopy images (first row: a, b, c) of the surface of the PS-*b*-PMMA-*SP* films prepared at a polymer concentration of 3 g/L without and with ZnS(NPs) at 5 wt.% before and after ultraviolet irradiation. The images in Figure 8 were measured at 20× magnification in the fluorescence microscope. The images show a structured and colored surface with blue and purple luminescence emission when the surface was exposed to visible light. These colors are related to the interaction of the photoactive polymer with ZnS (NPs) as was determined by UV-Vis spectrophotometry. At a concentration of 5 wt.% of ZnS (NPs), the films present spherical structures emitting yellow, blue and purple colors when exposed to natural light under the fluorescence optical microscope (Figure 8b). The blue color was related to the exciton through the band-gap of ZnS (NPs) [32] and the purple one is related to the presence of the photoactive moiety in the polymer as was determined by UV-Vis (see Figure 1). After UV irradiation (peak *λ* = 365 nm), the PS-*b*-PMAA-*SP*-ZnS (NPs) exhibits purple and blue luminescence as it is shown in Figure 8c. 

### 3.5. Band Gap Analysis

The optical band gap *E_g_* was estimated on a glass substrate from UV-Vis spectra before and after irradiation at 365 nm wavelength, using the Tauc plot [70]. The Tauc equation is:

(1) $$\alpha h\nu =A{\left(h\nu -E_{g}\right)}^{m}$$

where *α* is the absorption coefficient, *h* the Planck’s constant, *ν* frequency of light, *A* constant, *m* constant related to the type of optical transition and *E_g_* the band gap.

The absorption coefficient is estimated from *α* = 2.303 *A*/*d*. A is the absorbance and d is the compactness of the film [71]. The value of *m* = 1/2 was used in the calculations considering direct optical transition [32] in all compounds; therefore, (*αhν*)^2^ versus the *hν*, the photon energy, was plotted and the linear part of the figure was extrapolated to (*αhν*)^2^ = 0 to estimate the band gap. The estimated *E_g_* values are shown in Table 1 and the estimation procedure in Figures S5–S8.

The band gap determined for ZnS(NPs) (3.95 eV) is close to the maximum value reported [32] and exhibits a blue shift compared to literature due to the quantum confinement effect of nanocrystalline ZnS(NPs) thin films [72]. The band gap of ZnS and PS-*b*-PMMA-*SP* did not vary with the irradiation process. For the nanocomposite (PS-*b*-PMMA-*SP*-ZnS), after irradiation, the band gap decreased from 4.10 eV to 3.30 eV. Band gap reduction reflects the decreasing of the energy necessary for electrons to move from the valence to the conduction band.

While the band gap for the photoactive PS-*b*-PMMA-*SP* did not change after irradiation, it was reduced when combined with ZnS NPs to form PS-*b*-PMMA-*SP*-ZnS. The *E_g_* decreased from 4.20 to 4.10 eV, as shown in Table 1. This effect could be attributed to the attachment of *SP* moiety to the surface of the inorganic nanoparticles ZnS(NPs) on its ring-opened state. The coordination of charge negative ligands, i.e., either phenoxide or nitro moiety to the Zn^2+^ at the nanoparticle surface can increase the semiconductor HOMO band and therefore decrease the band gap for the development of a new transitional level beneath the conduction band [9].

As summarized in Table 1, after irradiation, the band gap of coordinated ZnS (NPs) is slightly greater than for photochromic agent (3.22 eV). Therefore, the purple luminescence was attributed to the interconversion of *MC* to *SP* of the photochromic agent grafted to the polymer matrix, which favors this balance when interacting with the ZnS (NPs). The incorporation of the ZnS (NPs) into the PS-*b*-PMMA-*SP* promotes the purple color emission (see Figure 8c) by changing the band gap value (see Table 1), but also decreases the light emission intensity after UV irradiation as shown in Figure 6b. The lower light intensity may be related to the energy transfer between the NPs and the photoactive polymer as found in Section 3.3 from emission measurements. The resulting PS-*b*-PMMA-*SP*-ZnS has a smaller band gap (3.30 eV) than the P(S)-*b*-PMAA-*SP* (4.20 eV).

## 4. Conclusions

This work reports the preparation of a photoactive polymer as a photo-switchable polymeric system in the presence and absence of ZnS nanoparticles and the influence of UV light irradiation on its optical properties. The presence of ZnS NPs increases the intensity ratio of –C–H stretching of methine with respect to those in methylene and increases the intensity of the aromatic –C–H stretching at 1452 cm^−1^. Both features could indicate the opening of the rigid *SP* structure into the *MC* form due to its interaction with the nanoparticle surface. The incorporation of ZnS NPs into the polymer avoids the self-assembling of the polymer as determined by SEM micrograph. This result is attributed to the interaction of the polymer with the surface of the nanoparticles. The optical properties of the polymer change upon incorporation of the nanoparticles. The overlaps of the absorption band of the polymer in the *MC* form with the emission band of ZnS NPs suggested an electron transfer mechanism from the *MC* to the nanoparticles upon irradiating the polymer with UV light. This effect was corroborated by fluorescence spectroscopy in which a decrease in the intensity with time in the of the fluorescence spectrum of the composite polymer was observed. Therefore, this work can contribute to the development of innovative functional and nanostructured materials with photosensitive character, since photosensitive molecular switches can be applicable in the development optical technological devices.

## Figures and Tables

**Table 1:** Optical band gap *E_g_* estimated from Tauc plot ^1^.

Case	*E_g_* (eV)	
	Before Irradiation	After Irradiation
ZnS	3.95	3.95
PS-*b*-PMMA-*SP*	4.20	4.20
PS-*b*-PMMA-*SP*–ZnS	4.10	3.30
6-nitrobenzopyran (*SP*)	3.20	3.22

^1^ The band gap (*E*_g_) was estimated before and after irradiation at 365 nm [70].

## Data Availability

All the data are available within the manuscript.

## Supplementary Materials

The following are available online at https://www.mdpi.com/2073-4360/14/5/945/s1: Figure S1: X-ray diffraction pattern of ZnS (blende and hexagonal structures), Figure S2: Synthesis reaction of 1-(2-hydroxyethyl)-2,3,3-trimethylindolenine bromide, Figure S3: Synthesis of 1-(2-hydroxyethyl)-3,3-dimethylindoline-6-nitrobenzopyran, Figure S4: (**a**) ^1^H-NMR spectrum of 1-(2-hydroxyethyl)-3,3-dimethylindoline-6-nitrobenzopyran (**b**) FT-IR of 1-(2-hydroxyethyl)-3,3-dimethylindoline-6-nitrobenzopyran, Figure S5: Band gap of ZnS (**a**) before and (**b**) after ultraviolet irradiation (365 nm, 0.4 mW/cm^2^), Figure S6: Band gap of 1-(2-hydroxyethyl)-3,3-dimethylindoline-6-nitrobenzopyran (*SP*) (**a**) before and (**b**) after ultraviolet irradiation (365 nm, 0.4 mW/cm^2^), Figure S7: Band gap of PS-*b*-PMMA-*SP* (**a**) before and (**b**) after ultraviolet irradiation (365 nm, 0.4 mW/cm^2^ for 10 min), Figure S8: Band gap of PS-*b*-PMMA-*SP*-ZnS (NPs) (**a**) before and (**b**) after ultraviolet irradiation (365 nm, 0.4 mW/cm^2^ for 10 min).
