# Supplementary material for: Design and Study of a Photo-Switchable Polymeric System in the Presence of ZnS Nanoparticles under the Influence of UV Light Irradiation
Source: Polymers (Basel). 2022 Feb 26;14(5):945. doi: 10.3390/polym14050945 (PMC8912439; doi:10.3390/polym14050945)
Supplement: Supplementary file 1 [file polymers-14-00945-s001.zip › polymers-1530649-supplementary.pdf]

polymers

MDPI

Supplementary Material

Design and Study of a Photo-Switchable Polymeric System in the Presence of ZnS Nanoparticles under the Influence of UV Light Irradiation

Guadalupe del C. Pizarro [1,*], Wilson Alavia [1,2], Karen González [1], Héctor Díaz [1], Oscar G. Marambio [1], Rudy Martin-Trasanco [1], Julio Sánchez [3], Diego P. Oyarzún [4] and Andrónico Neira-Carrillo [5]

[1] Departamento de Química, Facultad de Ciencias Naturales, Matemáticas y Medio Ambiente, Universidad Tecnológica Metropolitana, Santiago 7800003, Chile; w.alaviam@outlook.com (W.A.); karen.005@hotmail.es (K.G.); hdiaz@utem.cl (H.D.); omarambi@gmail.com (O.G.M.); rudy.martint@utem.cl (R.M.-T.)
[2] Programa Institucional de Fomento a la Investigación, Desarrollo e Innovación (PIDi), Universidad Tecnológica Metropolitana, Santiago 7800003, Chile
[3] Departamento de Ciencias del Ambiente, Facultad de Química y Biología, Universidad de Santiago de Chile, USACH, Santiago 9170022, Chile; julio.sanchez@usach.cl
[4] Laboratorio de Nanotecnología, Recursos Naturales y Sistemas Complejos, Facultad de Ciencias Naturales, Universidad de Atacama, Copiapó 1530000, Chile; diequim@gmail.com
[5] Departamento de Ciencias Biológicas, Facultad de Ciencias Veterinarias y Pecuarias, Universidad de Chile, Santiago 7800003, Chile; aneira@uchile.cl
* Correspondence: gpizarro@utem.cl or pizarroguadalupe048@gmail.com; Tel.: +56-2-27877132

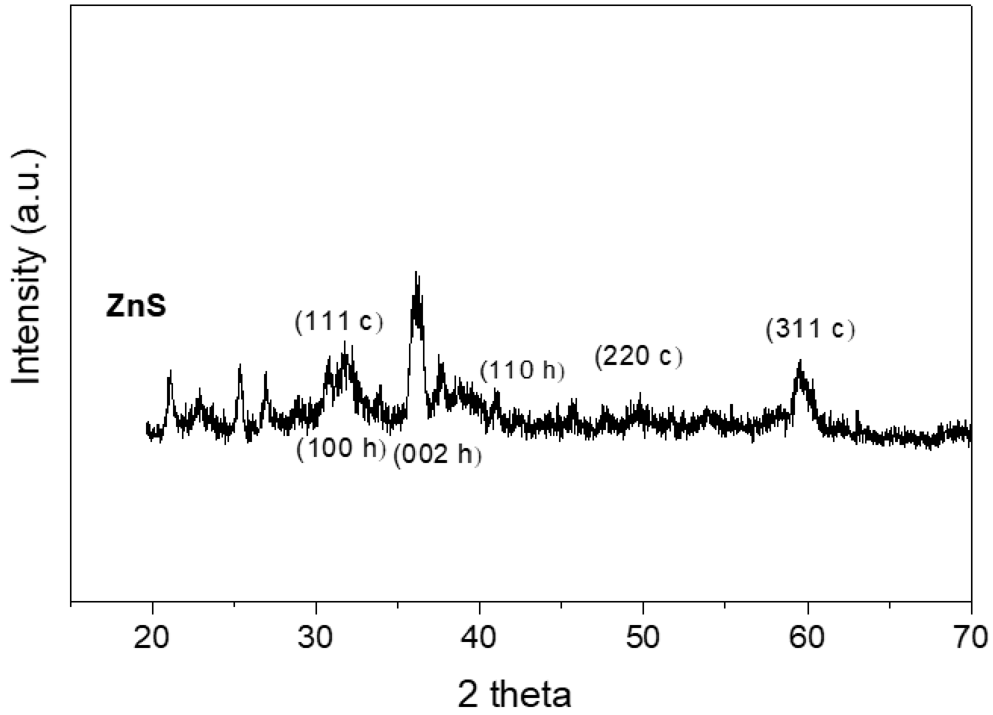

Figure S1. X-ray diffraction pattern of ZnS (blende and hexagonal structures).

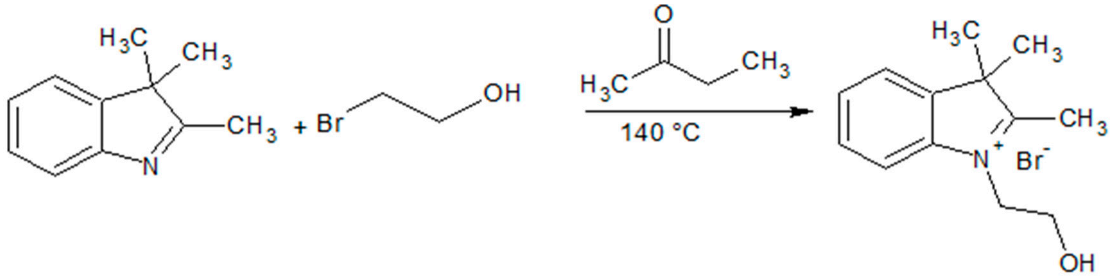

Figure S2. Synthesis reaction of 1- (2-hydroxyethyl) -2,3,3-trimethylindolenine bromide.

Figure S3 a) shows the following chemical shifts at (δ, ppm, $CDCl_3$): 7.57 (4H, multiplet, aromatic ring); 4.89 (2H, triplet, CH_2-N); 4.20 (2H, triplet, CH_2-OH); 3.15 (1H, singlet, -OH); 1.65 (9H, singlet, -CH_3-). The signals and displacements shown in the 1 H-NMR spectrum confirm the molecular structure of 1- (2-hydroxyethyl) -2,3,3-trimethylindolenine bromide.

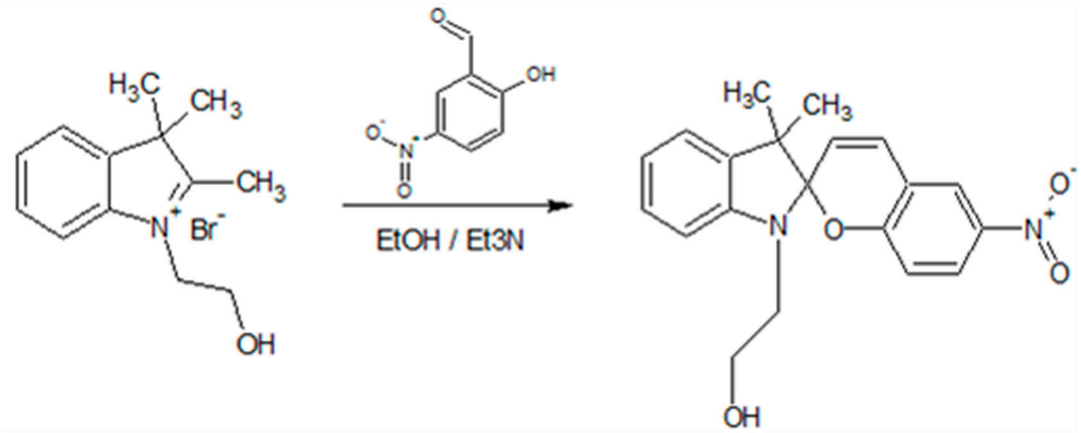

Figure S3. Synthesis of 1- (2-hydroxyethyl) -3,3-dimethylindoline-6- nitrobenzopyran.

^{1}HNMR (δ, ppm, $CDCl_3$) spectrum for the 1- (2-hydroxyethyl) -3,3-dimethylindoline-6-nitrobenzopyran exhibits clearly characteristic resonance signals, which confirmed the structure of the compound (see Figure S4), at: 7.97-7.92 [2H, multiplet, H (a) and H (b)]; 7.18 [1H, triplet, H (c)]; 7.01 [1H, doublet, H (d)]; 6.84 [2H, triplet, H (e) & H (f)]; 6.71 [1H, doublet, H (g)]; 6.57 [1H, doublet, H (h)]; 5.84 [1H, doublet, H (i)]; 3.71 [2H, triplet, CH_2-OH]; 3.46 [2H, multiplet, CH_2-N]; 1.53 [1H, singlet, -OH]; 1.22 [3H, singlet, -CH_3-]; 1.12 [3H, singlet, -CH_3-]. The FT-IR spectra (cm^{-1}, KBr) shows the following vibrational bands at 3365 cm^{-1} (O-H stretch; -CH_2OH); 3068 cm^{-1} (stretching =C-H); 2961 cm^{-1} (stretching CH-, -CH_2-, -CH_3); 1930-1836 cm^{-1} (Ar-H aromatic overtones); 1603 cm^{-1} (stretching -C=C-); 1510 cm^{-1} (asymmetric stretching Ar-NO_2); 1335 cm^{-1} (symmetric stretch Ar-NO_2); 1088 cm^{-1} (flexion -C-O-C-). The signals are characteristic of the photochromic compound 1-(2-hydroxyethyl)-3,3-dimethylindoline-6- nitrobenzopyran, (see Figure S4).

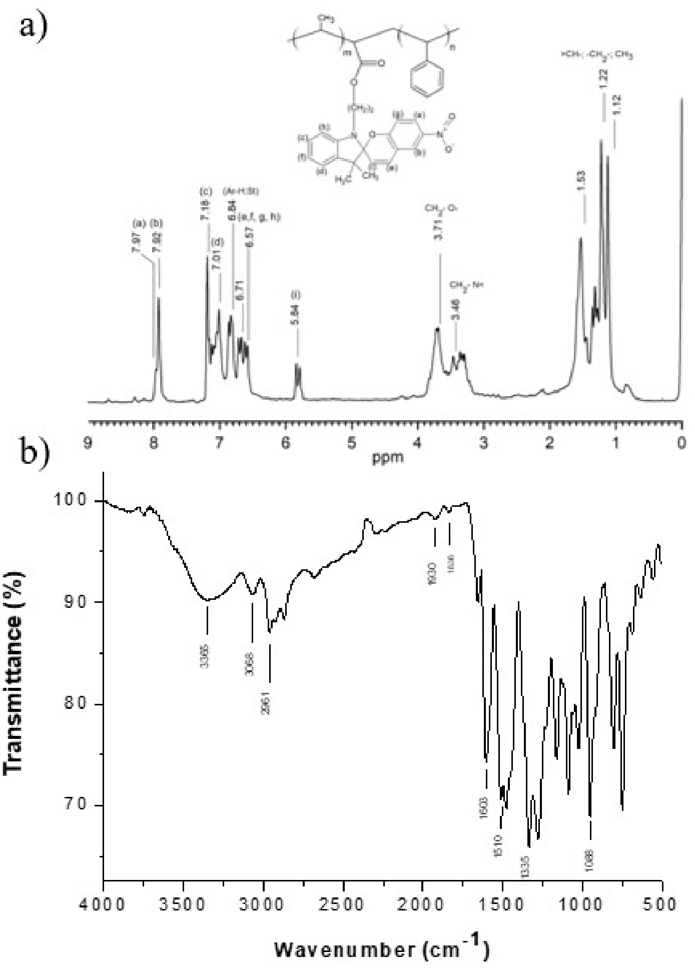

Figure S4. (**a**) ^{1}H-NMR spectrum of 1- (2-hydroxyethyl) -3,3-dimethylindoline-6-nitrobenzopyran (**b**) FT-IR of 1- (2-hydroxyethyl) -3,3-dimethylindoline-6-nitrobenzopyran.

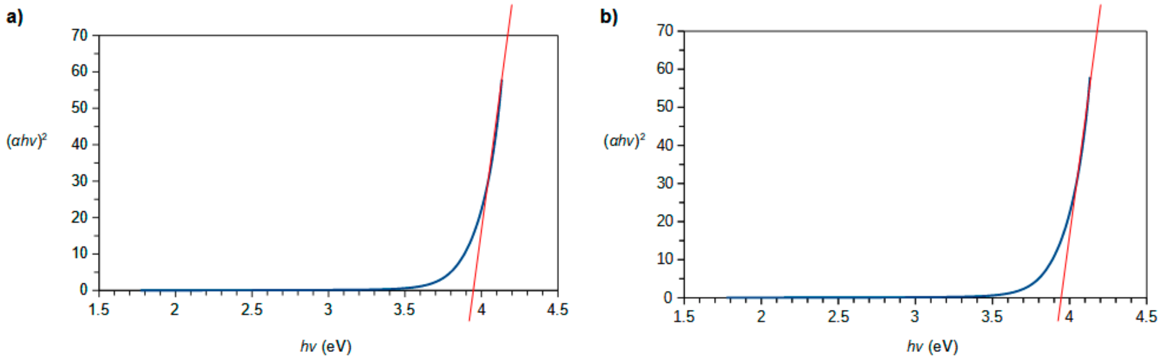

Figure S5. Band gap of ZnS (**a**) before and (**b**) after ultraviolet irradiation (365 nm, 0.4 mW/ cm^2).

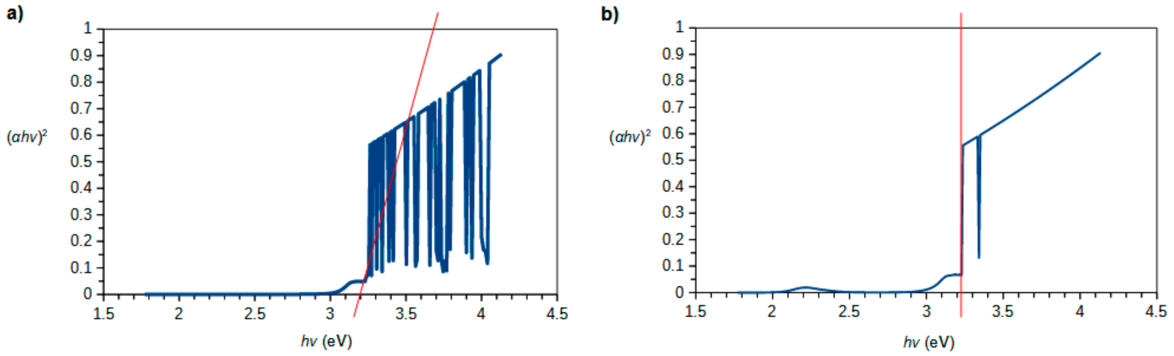

Figure S6. Band gap of 1-(2-hydroxyethyl)-3,3-dimethylindoline-6-nitrobenzopyran (*SP*) (**a**) before and (**b**) after ultraviolet irradiation (365 nm, 0.4 mW/cm^2).

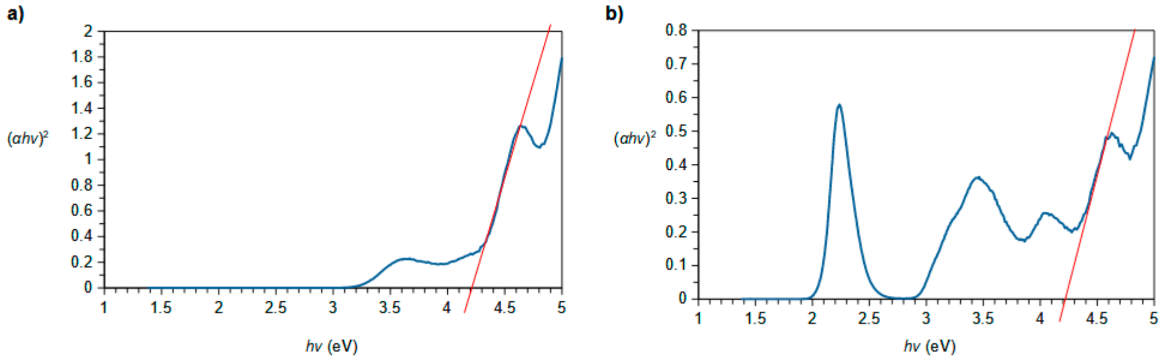

Figure S7. Band gap of PS-*b*-PMMA-***SP*** (**a**) before and (**b**) after ultraviolet irradiation (365 nm, 0.4 mW/cm^2 for 10 min).

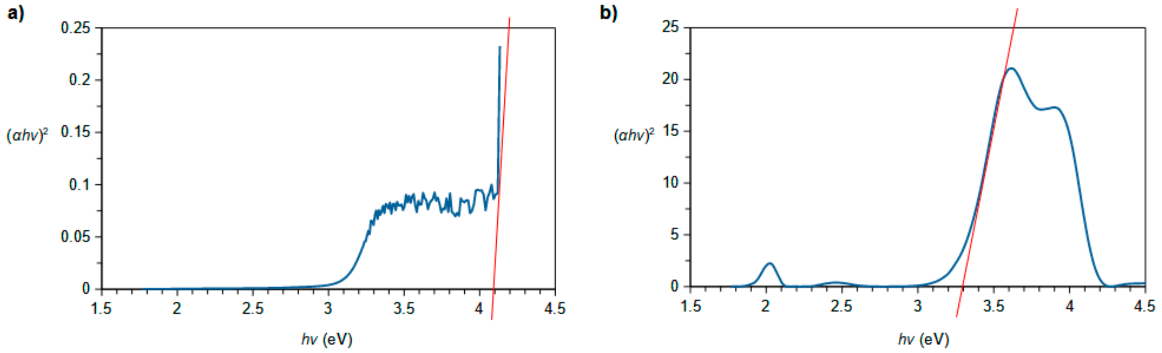

Figure S8. Band gap of PS-*b*-PMMA-***SP***-ZnS (NPs) (**a**) before and (**b**) after ultraviolet irradiation (365 nm, 0.4 mW/cm^2 for 10 min).
